# The effectiveness of a computer-aided system in improving the detection rate of gastric neoplasm and early gastric cancer: study protocol for a multi-centre, randomized controlled trial

**DOI:** 10.1186/s13063-023-07346-5

**Published:** 2023-05-11

**Authors:** Zehua Dong, Yijie Zhu, Hongliu Du, Junxiao Wang, Xiaoquan Zeng, Xiao Tao, Ting Yang, Jiamin Wang, Mei Deng, Jun Liu, Lianlian Wu, Honggang Yu

**Affiliations:** 1 Department of Gastroenterology, Renmin Hospital of Wuhan University, Wuhan, Hubei China; 2 Key Laboratory of Hubei Province for Digestive System Disease, Renmin Hospital of Wuhan University, Wuhan, China; 3 Hubei Provincial Clinical Research Center for Digestive Disease Minimally Invasive Incision, Renmin Hospital of Wuhan University, Wuhan, China

**Keywords:** Artificial intelligence, Early gastric cancer, Randomised controlled trial

## Abstract

**Background:**

This protocol is for a multi-centre randomised controlled trial to determine whether the computer-aided system ENDOANGEL-GC improves the detection rates of gastric neoplasms and early gastric cancer (EGC) in routine oesophagogastroduodenoscopy (EGD).

**Methods:**

*Study design*: Prospective, single-blind, parallel-group, multi-centre randomised controlled trial.

*Settings*: The computer-aided system ENDOANGEL-GC was used to monitor blind spots, detect gastric abnormalities, and identify gastric neoplasms during EGD.

*Participants*: Adults who underwent screening, diagnosis, or surveillance EGD.

Randomisation groups:

1. Experiment group, EGD examinations with the assistance of the ENDOANGEL-GC;

2. Control group, EGD examinations without the assistance of the ENDOANGEL-GC.

*Randomisation*: Block randomisation, stratified by centre.

*Primary outcomes*: Detection rates of gastric neoplasms and EGC.

*Secondary outcomes*: Detection rate of premalignant gastric lesions, biopsy rate, observation time, and number of blind spots on EGD.

*Blinding*: Outcomes are undertaken by blinded assessors.

*Sample size*: Based on the previously published findings and our pilot study, the detection rate of gastric neoplasms in the control group is estimated to be 2.5%, and that of the experimental group is expected to be 4.0%. With a two-sided α level of 0.05 and power of 80%, allowing for a 10% drop-out rate, the sample size is calculated as 4858. The detection rate of EGC in the control group is estimated to be 20%, and that of the experiment group is expected to be 35%. With a two-sided *α* level of 0.05 and power of 80%, a total of 270 cases of gastric cancer are needed. Assuming the proportion of gastric cancer to be 1% in patients undergoing EGD and allowing for a 10% dropout rate, the sample size is calculated as 30,000. Considering the larger sample size calculated from the two primary endpoints, the required sample size is determined to be 30,000.

**Discussion:**

The results of this trial will help determine the effectiveness of the ENDOANGEL-GC in clinical settings.

**Trial registration:**

ChiCTR (Chinese Clinical Trial Registry), ChiCTR2100054449, registered 17 December 2021.

**Supplementary Information:**

The online version contains supplementary material available at 10.1186/s13063-023-07346-5.

## Introduction

Gastric cancer is the fourth leading cause of cancer-related deaths, with an estimated 1,080,000 new cases and 760,000 deaths in 2020 [1]. Early gastric cancer (EGC) is generally asymptomatic; therefore, most patients are not diagnosed until the disease has progressed to an advanced stage, with a 5-year survival rate of < 25% [2]. Early detection is vital to improve the prognosis of patients with gastric cancer [3].

Upper gastrointestinal endoscopy is widely used to detect and diagnose early gastric cancers [4]. White light endoscopy (WLE) is the current standard and most commonly used tool [5]. However, the diagnostic performance of WLE is not satisfactory, with a pooled sensitivity of 48.0% (95% confidence interval [CI], 39.0–57.0%) and a pooled specificity of 67.0% (95% CI, 62.0–71.0%) [6]. To improve diagnostic ability, new techniques such as image-enhanced and magnifying endoscopy have been proposed [7, 8]. However, the high cost of equipment, complex diagnostic theories, and strict training requirements for endoscopists limit the popularity of these techniques. Thus, the findings of this study are of great importance and have the potential to improve the diagnostic ability of WLE.

Despite its diagnostic ability, the quality of endoscopic examination plays an important role in the detection of EGC, which is a prerequisite for lesion detection [9]. To ensure endoscopic quality, guidelines and expert consensus have prescribed and advocated the full examination of the entire stomach [10, 11]. However, these recommendations are not well implemented in clinical practice because of insufficient practical quality control tools and strong supervision [12]. Li et al. reported that completeness of stomach screening was positively related to the detection rate of early cancers. Therefore, it is vital to ensure the completeness of the examinations [13].

With the rapid progress in artificial intelligence (AI) technology, several researchers have developed intelligent systems aimed at diagnosing EGC or upper gastrointestinal neoplasms, and few studies have aimed to ensure the completeness of examinations [14, 15]. In 2019, we first applied deep learning algorithms to the field of endoscopy quality control. We developed WISENSE (renamed ENDOANGEL), an intelligent system aimed at monitoring blind spots in upper gastrointestinal endoscopy in real time [16]. Single-centre and multi-centre randomised controlled trials (RCTs) have proven to significantly reduce the blind spot rate in examinations. Furthermore, we developed ENDOANGEL-LD (lesion detection) to detect gastric abnormalities and diagnose gastric neoplasms (low-grade dysplasia, gastric adenomas, gastric lymphomas, EGC, and advanced gastric cancers) in real time [17]. In another single-centre, tandem-designed trial, we proved that ENDOANGEL-LD could reduce the biopsy rate and miss rates of gastric neoplasms in real time [18].

Development of a tool with the ability to simultaneously improve the diagnostic performance of WLE and ensure high completeness of examination is an ideal way to fulfil these needs. Therefore, we proposed the AI system ENDOANGEL-GC (gastric cancer), which simultaneously combines the functions of blind spot monitoring and lesion detection and runs in real time. We designed this randomised, controlled, patient-blinded, multi-centre trial with two parallel groups and a 1:1 allocation to evaluate the hypothesis that ENDOANGEL-GC would improve the detection rates of gastric neoplasms and EGC.

## Methods/design

### Study design

**Fig. 1:**
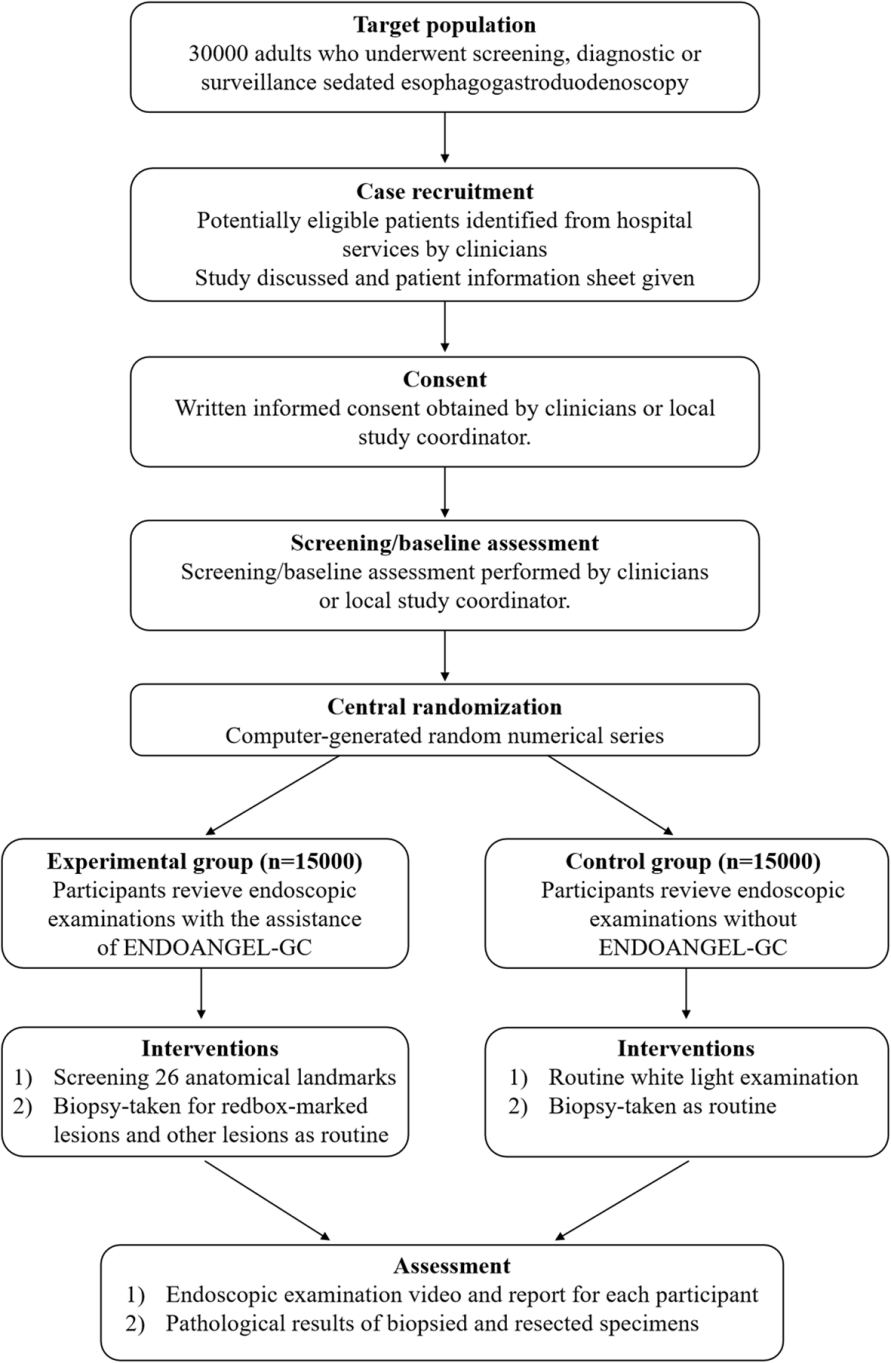
Study design of this trial

**Fig. 2:**
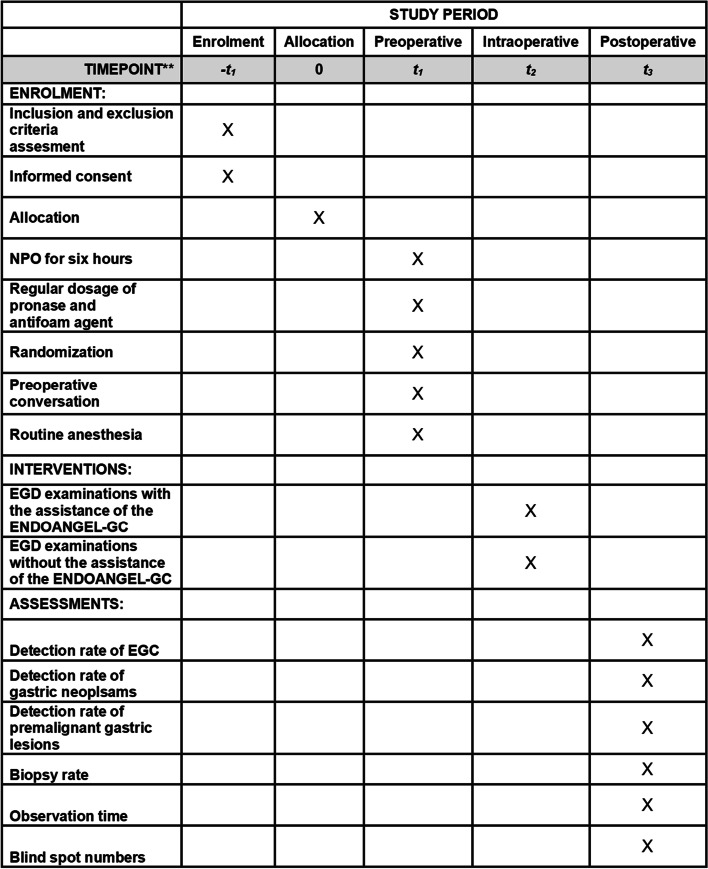
The SPIRIT schedule of enrolment, interventions, and assessments

This is a prospective, single-blind, parallel-group, multicentre, RCT. The recruited participants will be randomised into either the experimental or control groups at a ratio of 1:1. Participants in the experimental group will undergo endoscopic examinations with the assistance of ENDOANGEL-GC, while those in the control group will undergo routine examinations without ENDOANGEL-GC feedback. The study design is illustrated in Fig. 1. This study will be performed according to the Standard Protocol Items: Recommendations for Interventional Trials (SPIRIT) [19]. The SPIRIT Checklist is provided in the supplementary materials. Figure 2 shows the SPIRIT schedule for enrolment, intervention, and assessment.

### Study setting

This study is being conducted in large-scale, primary hospitals in China.

### Participants and recruitment

Participants who fulfil the following criteria are eligible:

Inclusion criteria

- Aged ≥ 18 years
- Aim to undergo screening, surveillance, and diagnosis
- Undergo sedated EGD
- Able to read, understand, and sign informed consent

Exclusion criteria

- EGD contraindications
- Not suitable for sedated endoscopy after anaesthesia evaluation
- Biopsy contraindications
- Active upper gastrointestinal bleeding or emergency oesophagogastroduodenoscopy (EGD)
- Pregnancy
- Upper gastrointestinal surgery or residual stomach
- Not suitable for recruitment after investigator evaluation because of other high-risk conditions

## Interventions

### Procedures

The ENDOANGEL-GC is an AI system designed for assisting in EGD and possesses three functions in real time: (1) to time the entire procedure (from the endoscope intubating into the mouth to it being drawn out), (2) to record observed upper gastrointestinal tract landmarks and blind spots, and (3) to mark the upper gastrointestinal lesion with a blue rectangular box (box colour turns red if the lesion is predicted to have a high risk of gastric neoplasm) [16, 17].

The ENDOANGEL-GC is connected to the endoscopy processor, receives digital images as input, and outputs the predictions of landmark monitoring and lesions. The ENDOANGEL-GC is installed on a separate computer system, and the output of the system appears on the same monitor as that of the endoscope. A button on the system is set to switch between “assistance” and “no assistance” modes. Before examination in the control group, the ENDOANGEL-GC is shifted to the “no assistance” mode, so the predictions will be concealed, as they are not displayed on the monitor. Before the examination in the experimental group, the ENDOANGEL-GC is shifted to the “assistance” mode, so the predictions and prompts are displayed on the screen.

The endoscopists who participate in the study are required to have EGD experience with more than 500 cases. All participating endoscopists will be trained in the AI’s functions, including monitoring anatomical landmarks, detecting suspicious lesions, and becoming familiar with the operation requirements during endoscopy. Each endoscopist will perform at least 10 examinations with AI assistance. In both groups, endoscopists are required to screen the entire stomach under white light mode. Only when the screening process is completed and suspicious lesions are found, the image-enhanced mode will be allowed for further judgement. In the experimental group, the endoscopists will operate the endoscope with the assistance of ENDOANGEL-GC. They are required to adhere to the following criteria: (1) screen anatomical landmarks according to the feedback of ENDOANGEL-GC and (2) lesions marked in red boxes by ENDOANGEL-GC are required to undergo biopsy sampling. Lesions that are not marked in red boxes but meet any of the following criteria are also recommended for biopsy: differences in colour, loss of vascularity, slight elevation or depression, nodularity, thickening, abnormal convergence or flattening of folds, irregular margins, irregular discoloration, or irregular surfaces. In the control group, endoscopists will routinely operate on the endoscope. Lesions that meet the above criteria are also recommended for biopsy.

### Adherence and protocol deviations

To enhance the validity of the data, face-to-face adherence reminder sessions and a pilot study will be conducted before enrolment at each study site. Additionally, a key method will be followed for assessing adherence. All raw videos of the examinations, with and without the ENDOANGEL-GC dashboard, will be recorded, stored, and reviewed.

The following conditions will be defined as protocol deviations in the experimental group and will be excluded from the per-protocol (PP) population: (1) more than two anatomical landmarks not observed after video review and (2) biopsy sampling or further treatment not performed for red-box lesions with no pathology results for such lesions.

The following conditions were defined as protocol deviations in both the experimental and control groups and were excluded from the PP population: (1) the inclusion and exclusion criteria are determined to be unfulfilled after the participant is randomised; (2) inability to fully observe the gastric anatomical landmarks due to gastric retention, residual stomach, or oesophageal obstruction; and (3) incorrect mode of ENDOANGEL-GC.

## Recruited cases

### Consent

Patients eligible to participate in this study will be provided with further discussions and informed consent. Discussions may be completed by a local study coordinator or a staff member. Written informed consent will be obtained from all participants who agree and wish to take part in the study.

### Eligibility and baseline assessment

Once written informed consent is obtained, an eligibility assessment will be performed by the local study coordinator or staff, according to the inclusion/exclusion criteria. For each eligible participant, the following baseline characteristics will be collected: gender; age; weight; height; indications; education; nationality; registered residence (rural or urban); source (inpatient or outpatient); permanent address; *H. pylori* infection status; history of smoking and drinking; history of hypertension, diabetes, and coronary heart disease; and family history of cancer (especially oesophageal and stomach cancer). The eligibility and baseline assessment procedures will be conducted using a mobile device (tablet or phone) and web-based electronic data capture (EDC) system.

### Recruited case management

All recruited participants will be assigned identifications (IDs) comprising eight codes in the EDC system. The first four codes represent different hospitals, whereas the last four codes represent the recruiting sequences. This ID will not be used repeatedly.

### Pre-, intra-, and postoperative management

*Preoperative management* is as follows: (1) NPO for 6 h, (2) regular dosage of pronase and antifoam agent taken before examinations, (3) randomisation, (4) preoperative conversation, and (5) routine anaesthesia.

*Intraoperative management* is as follows: (1) positional nursing, lateral position; (2) insertion; (3) general method for screening upper gastrointestinal tract; (4) tissue sampling (if needed), specimen handing, and laboratory processing, and specimens will be sent to the pathology department of local study centres; (5) routine intraoperative monitoring and first-aid package preparation; and (6) intraoperative endoscopic photo documentation and report records.

*Postoperative management* is as follows: (1) routine postoperative treatment and nursing, (2) observation of vital signs in the recovery room, and (3) adverse events.

### Specimen management and pathological traceability

After tissue sampling, the specimens that arrive in the pathology department will be dehydrated, embedded, sliced, and stained. Pathological results will be obtained from local study centres by expert pathologists at each centre. Regarding patients diagnosed with gastric cancers by biopsy and those highly suspected with gastric cancer without biopsy who will possibly take further elective operations, their pathological results of resected specimens will be traced for up to 60 days from their endoscopy date to determine the invasion depth of gastric cancer.

After the training program, a group of five research assistants will watch the raw video of each participant and capture clean, high-quality frames of the lesions with pathological results under the guidance of an expert endoscopist. All images of the lesions and corresponding pathological results will be sent to an independent group of three expert endoscopists for further endoscopic evaluation. Randomisation and personal information will be concealed, and the experts will be unaware of the allocation of the lesions. A group of three experts will evaluate the endoscopic and pathological diganosis of a lesion and determine whether they are unmatched. (Here was a clerical error of describing the determination of the unmatched lesions, and we have corrected it). De-identified digital pathological slides of unmatched lesions will be sent to an independent group of expert pathologists for consultation. Only unmatched lesions will be reviewed by an independent group of expert pathologists for the final diagnosis.

Each expert pathologist is required to determine whether the slide contains acute inflammation, chronic inflammation, atrophy, or intestinal metaplasia and to rate the severity (mild, moderate, or severe) of these pathological changes. In addition, they are required to determine the presence of low-grade intraepithelial neoplasia, high-grade intraepithelial neoplasia, or carcinoma. Multiple pathological changes may occur in the same lesion, the most severe being the primary diagnosis. The severity of the pathological changes increases from inflammation to carcinoma. A lesion is diagnosed only when two or more experts reach a consensus. If the three expert pathologists did not reach a consensus, the lesion was re-evaluated until a consensus was reached.

## Randomisation

Local study coordinators or staff accomplished randomisation using an EDC system. Participants were randomised into either the experimental or control group at a 1:1 ratio before the examinations. The randomisation results, time points, and case IDs will be stored online in the EDC system.

Participants will be randomised into one of the two arms by block randomisation stratified by centre. The randomisation sequence will be developed using a computer-generated random numerical series, with one encoding for the AI-assisted group and zero encoding for the control group. The original sequence will be stored in an online central randomisation system database. The online sequence will not accessible to investigators or study coordinators. If a subject fulfils the enrolment criteria, the authorised study coordinators or staff will log in to the password-protected account to obtain the assignment for him/her.

## Blinding

Patients, pathologists, and data analysts will be blinded to the randomisation. Masking of study group allocations will not attempted by the endoscopists. Randomisation results will be concealed in information brochures or other documents for the participants.

The interventions in this study will not add additional risks to participants, compared with routine sedated EGD. However, if a patient has an unexpected adverse event unrelated to the intervention and requires disclosure of study assignment information, then unblinding can be performed.

## Outcomes

The primary outcome measures are detection rate of gastric neoplasms and EGC detection rate. The gastric neoplasm detection rate is defined as the ratio of patients with neoplasms to the recruited population. The EGC detection rate is defined as the ratio of patients with EGC to all patients with gastric cancer. EGC includes pathologically proven high-grade intraepithelial neoplasia and gastric cancer restricted to the mucosa and submucosa.

Secondary outcome measures include the detection rate of premalignant gastric lesions, biopsy rate, observation time, and number of blind spots on EGD examination. The detection rate of premalignant gastric lesions was defined as the ratio of patients with premalignant gastric lesions to the entire recruited population. The biopsy rate is defined as the ratio of patients who undergo biopsy in the recruited population. The observation time is defined as the overall examination time under WLE minus the biopsy operation time. Blind spots are defined as unobserved landmarks.

## Study withdrawal

Eligible participants will be included in this study after providing informed consent and undergoing randomisation. The participants can withdraw at any time during the study. Data collected prior to withdrawal can be used in this study if informed consent is obtained. Participants should be withdrawn in the following situations: (1) perforation, (2) massive haemorrhage, (3) allergy to sedative medication, (4) poor preparation, and (5) gastric retention. Withdrawn participants will not be replaced by other recruited participants after they sign the informed consent form.

## Safety evaluation

Adverse events are evaluated according to the Common Terminology Criteria for Adverse Events [20]. Occurrence time, expiration time, interventions, and treatments will be recorded.

## Data analysis

### Sample size calculation

The sample size is calculated on the basis of the primary outcomes.

The detection rate of gastric neoplasms and EGC detection rate without AI assistance were determined by literature research. Zhang et al. reported an EGC detection rate of 20.0 to 20.9% [21] and a gastric neoplasm detection rate of 1.63%. Di et al. reported an EGC detection rate of 16.44% and a gastric neoplasm detection rate of 3.37% [22]. Therefore, the EGC detection rate in the control group is estimated to be 20%, and the gastric neoplasm detection rate is estimated to be 2.5% (the mean value of the two references).

According to our previous study, the detection rate of gastric neoplasms increased by 1.35% when assisted by an AI system; therefore, the detection rate of gastric neoplasms in the experimental group is estimated to be 4.0%. There are no published data evaluating the improvement in EGC detection rates with AI assistance. Thus, we referred to two studies reporting improvements of > 80% in EGC detection sensitivity and proportion [23, 24], resulting in an estimation that the EGC detection rate will increase from 20% to 35% with AI assistance.

The sample size was calculated, using the *Z* test method, as 4858 based on the detection rate of gastric neoplasms, with a two-sided *α* level of 0.05 and power of 80%, allowing for 10% drop-out rate. Regarding the sample size based on the EGC detection rate, the *Z* test method was used for calculation with a two-sided *α* level of 0.05 and power of 80%, resulting in a total of 270 cases of gastric cancer. Assuming the proportion of gastric cancer to be 1% in patients undergoing EGD and allowing for a 10% dropout rate, the sample size was calculated as 30,000. Considering the larger sample size calculated from the two primary endpoints, the required sample size was determined to be 30,000.

To achieve adequate participant enrolment, we first evaluated the annual volume of sedated EGD examinations at all participating centres. The sum of the examination volumes of all centres (> 20,000 per year) is sufficient to provide support for the completion of the study. In each centre, a study coordinator, group of nurses, or endoscopists will be pretrained for informed consent before the examination. Consecutive patients will be informed about the study and assessed for eligibility.

### Data collection

Data will be collected in a standard case-report form through the EDC system and anonymised for further analysis. Data include baseline information, endoscopic reports, and pathological results. Data will be de-identified before being entered into the database. Regular quality monitoring and database checking will be performed at each centre to ensure data accuracy.

In addition, the computer on which the ENDOANGEL-GC runs will be equipped with video-recording software. It is used to capture and store video signals from the endoscope device and predictions from the ENDOANGEL-GC system. For each examination, an unprocessed raw video from the endoscope and a video with ENDOANGEL-GC predictions (“AI” videos) are recorded and stored.

### Data analysis plan

The analysis will use intention-to-treat (ITT) and PP approaches. The ITT population will include all patients who are randomised, whereas the PP population will include patients who undergo EGD in accordance with the assigned intervention. The null hypothesis is that the detection rates of gastric neoplasms and EGC in the experimental group will not differ from those in the control group. An alternative hypothesis is that the detection rates of gastric neoplasms and EGC in the experimental group will differ from those in the control group. The experimental group (with ENDOANGEL-GC assistance) will be compared with the control group (without ENDOANGEL-GC assistance) for the two primary outcomes using the *χ*^2^ test.

Continuous variables will be expressed as means (standard deviations [SDs]) or medians (interquartile ranges [IQRs]), according to their distributions, and categorical variables will be presented as numbers (percentages). Baseline characteristics between the two groups will be compared using the *χ*^2^ test for categorical variables and the Mann-Whitney *U* test for continuous variables. Statistical significance is set at *p* < 0.05. A 95% confidence interval will be calculated. Subgroup analysis will be conducted, stratified by multiple indices, such as age and ENDOANGEL-GC-marked red boxes, indicating that only red-box-marked lesions will be included in the analysis. The histological data and lesion characteristics will be compared with the ENDOANGEL-GC prediction, and the performance of ENDOANGEL-GC will be further analysed.

In this study, the potential missing data include the following: (1) characteristic information (the missed data will be marked with “NA” and will not be included in the analysis); (2) pathological results, the gold standards for analysis (participants with missing pathological results are excluded from the analysis); and (3) original videos (the data contained information on whether the endoscopic procedure adhered to the protocol requirements). Participants without this information will be excluded from the per-protocol analyses.

## Dissemination of results

The data in this study are the properties of the chief investigator and the other co-investigators. This publication is the responsibility of the chief investigator. All co-investigators will have access to anonymised trial data for further analysis and publication of peer-reviewed journal articles.

## Study monitoring

Research assistants of chief investigating centre are responsible for regular study monitoring.

## Discussion

This study will explore the effectiveness of the AI system ENDOANGEL-GC in improving the detection rate of gastric neoplasms and EGC detection rate. We plan to enrol 30,000 participants from > 20 large-scale primary digestive centres in China. Enrolment began in December 2021. At the time of manuscript preparation, more than 10,000 patients had been enrolled. The results of this large, multi-centre RCT will provide high-level evidence for the application of AI systems in clinical settings.

## Trial status

The enrolment of this study is ongoing at the time of manuscript submission, adhering to the protocol with version 3.0 (July 5, 2021). Recruitment began on December 21, 2021, and is estimated to be completed on December 20, 2024.

## Roles and responsibilities

The principal investigator and research physician have contributed the following: designing and conducting of the trial, preparation of the protocol and revisions, and publication of study reports.

There were no trial steering committee or data monitoring committee in this trial.

## Supplementary Information

**Additional file 1.**

## Data Availability

All principal investigators will be given access to the cleaned datasets. All datasets will be password protected. Project principal investigators will have direct access to their own site’s datasets and will have access to other sites data by request. The data in this study are available from the corresponding author on reasonable request. The study protocol is accessible in this manuscript and on the registration website. The statistical software is publicly available.

## Abbreviations

ChiCTR: Chinese Clinical Trial Registry
GC: Gastric cancer
EGD: Esophagogastroduodenoscopy
EGC: Early gastric cancer
WLE: White light endoscopy
AI: Artificial intelligence
RCT: Randomized controlled trial
LD: Lesion detection
SPIRIT: Standard Protocol Items: Recommendations for Interventional Trials
GI: Gastrointestinal
EDC: Electronic data capture
ID: Identification
NPO: Nil per os
SD: Standard deviation
IQR: Interquartile range
